# Spherical Harmonics Reveal Standing EEG Waves and Long-Range Neural Synchronization during Non-REM Sleep

**DOI:** 10.3389/fncom.2016.00059

**Published:** 2016-06-22

**Authors:** Siddharth S. Sivakumar, Amalia G. Namath, Roberto F. Galán

**Affiliations:** Department of Electrical Engineering and Computer Science, School of Engineering, Case Western Reserve UniversityCleveland, OH, USA

**Keywords:** stage 2 sleep, spindles, sigma waves, volume conduction theory, agenesis, consciousness

## Abstract

Previous work from our lab has demonstrated how the connectivity of brain circuits constrains the repertoire of activity patterns that those circuits can display. Specifically, we have shown that the principal components of spontaneous neural activity are uniquely determined by the underlying circuit connections, and that although the principal components do not uniquely resolve the circuit structure, they do reveal important features about it. Expanding upon this framework on a larger scale of neural dynamics, we have analyzed EEG data recorded with the standard 10–20 electrode system from 41 neurologically normal children and adolescents during stage 2, non-REM sleep. We show that the principal components of EEG spindles, or sigma waves (10–16 Hz), reveal non-propagating, standing waves in the form of spherical harmonics. We mathematically demonstrate that standing EEG waves exist when the spatial covariance and the Laplacian operator on the head's surface commute. This in turn implies that the covariance between two EEG channels decreases as the inverse of their relative distance; a relationship that we corroborate with empirical data. Using volume conduction theory, we then demonstrate that superficial current sources are more synchronized at larger distances, and determine the characteristic length of large-scale neural synchronization as 1.31 times the head radius, on average. Moreover, consistent with the hypothesis that EEG spindles are driven by thalamo-cortical rather than cortico-cortical loops, we also show that 8 additional patients with hypoplasia or complete agenesis of the corpus callosum, i.e., with deficient or no connectivity between cortical hemispheres, similarly exhibit standing EEG waves in the form of spherical harmonics. We conclude that spherical harmonics are a hallmark of spontaneous, large-scale synchronization of neural activity in the brain, which are associated with unconscious, light sleep. The analogy with spherical harmonics in quantum mechanics suggests that the variances (eigenvalues) of the principal components follow a Boltzmann distribution, or equivalently, that standing waves are in a sort of “thermodynamic” equilibrium during non-REM sleep. By extension, we speculate that consciousness emerges as the brain dynamics deviate from such equilibrium.

## Introduction

Modern philosophy and science attribute the most significant aspects of the human mind, such as consciousness, to neural activity in the brain (Tononi and Edelman, 1998; McFadden, 2002; Rees et al., 2002; Libet, 2004; Lamme, 2006; Melloni et al., 2007; Fingelkurts et al., 2013). This conception inevitably leads scientists to pursue a better understanding of the brain as a complex physical system, in the hope that even its most perceptible, material properties will ultimately explain the highest transcendent features of human cognition and the mind.

Macroscopic physical properties of brain activity, including the electromagnetic fields generated by neural activity, are known to change with the state of awareness (Walter et al., 1967; Lehmann et al., 2001; Cahn and Polich, 2006; Fingelkurts et al., 2012; Cicurel and Nicolelis, 2015). Technologies developed to assess these phenomena therefore reveal information about the state of an individual's awareness, including but not limited to stages of sleep, concentration, and motor activity, which can be registered non-invasively as scalp electroencephalogram (EEG) or magnetoencephalogram (MEG). Spatiotemporal EEG and MEG patterns are sensitive enough to allow for an effective discrimination between certain cognitive states such as alertness and drowsiness (Sing and Russo, 2007), and even phenotypes such as schizophrenia (Van Der Stelt and Belger, 2007), amnesia (Babiloni et al., 2010), dyslexia (Babiloni et al., 2012), and autism (García Domínguez et al., 2013; Pérez Velázquez and Galán, 2013). This suggests that despite their apparently random nature, spontaneous patterns of brain activity are well-structured both in space and time (Galán, 2008; Fingelkurts et al., 2010).

Our lab has previously demonstrated how brain circuit connectivity constrains the activity patterns displayed in brain circuits (Galán, 2008; Steinke and Galán, 2011). Specifically, we have shown that the principal components of spontaneous neural activity are uniquely determined by the underlying circuit connectivity, and that although those principal components do not uniquely resolve the circuit structure, they do reveal important features about their functional connectivity, such as the size of center-surround inhibition (Galán, 2008). More generally, it is well-known from geophysics and climate research that the principal components of spatiotemporal patterns of physical parameters, such as surface sea-water temperature or air pressure, represent stationary (i.e., non-propagating) oscillations, also known as standing waves (Storch and Zwiers, 2001). Standing waves are spatially constrained oscillations where each point over the spatial domain is associated with a constant maximum amplitude over time, giving rise to nodes where the amplitude is consistently zero. On a spherical domain, standing waves appear as spherical harmonics with multiple poles where the amplitude of the wave is maximized. By definition, the principal components identify spatial locations with a coherent fluctuating pattern, a property that can be used to detect stationary climate oscillations such as *El Niño* or the *North Atlantic Oscillation* (Storch and Zwiers, 2001).

In neuroscience, the existence of standing EEG waves was already predicted by early neural mass models of brain activity (Nunez, 1981), which have been expanded and refined ever since (Nunez, 1998; Nunez and Srinivasan, 2006a,b). Those models also predict that, since the human head conforms to a sphere, EEG signals can be mathematically expanded into a basis of spherical harmonics (Wingeier et al., 2001). However, to the best of our knowledge, no empirical evidence for physical waves in the form of multipolar spherical harmonics has been provided to date.

Sleep is often considered the true resting state of the brain, and it is when “the most synchronized network patterns occur” (Buzsaki and Watson, 2012), additionally lending ease to filtering and analysis (Campbell, 2009). One of the most recognizable features of EEG during sleep is the sleep “spindle,” or sigma wave (10–16 Hz), a characteristic phenomenon of non-rapid eye movement (non-REM) sleep that is hypothesized to mediate the process of memory consolidation (Schabus et al., 2004; Marshall and Born, 2007), and is observed as simultaneous short-lived wavelets across EEG channels (Andrillon et al., 2011; Bonjean et al., 2012; Niknazar et al., 2015). Stage 2 non-REM sleep is the most frequently employed stage for observing spindle activity, and is dominated by centroparietal waves (Ayoub et al., 2013), which are believed to be thalamo-cortical in nature (Bonjean et al., 2012; Niknazar et al., 2015).

In this context, we have investigated the principal components of EEG sigma waves during non-REM sleep in 41 children and adolescents with no neurological conditions. First, we show both mathematically and empirically that the principal components of these waves are spherical harmonics, which in turn represent standing waves on the head's surface. Second, in order to assess the thalamo-cortical nature of the spindles, we performed similar analyses on 8 children with complete absence of, or severe reduction in, inter-hemispherical cortico-cortical connections, also known as the *corpus callosum*. The principal components of sigma waves in these subjects are also spherical harmonics, including dipoles and quadrupoles across both hemispheres, suggesting that sleep spindles are mainly driven by coherent thalamo-cortical oscillations, rather than cortico-cortical oscillations. Third, using volume conduction theory, we demonstrate that current densities over the brain's surface can be computed in a straightforward manner for standing EEG waves, which subsequently allow us to define a characteristic length for large-scale neural synchronization. Finally, we speculate about the role of standing EEG waves in the conscious and unconscious brain.

## Results

### Overview of EEG data

**Figure 1:**
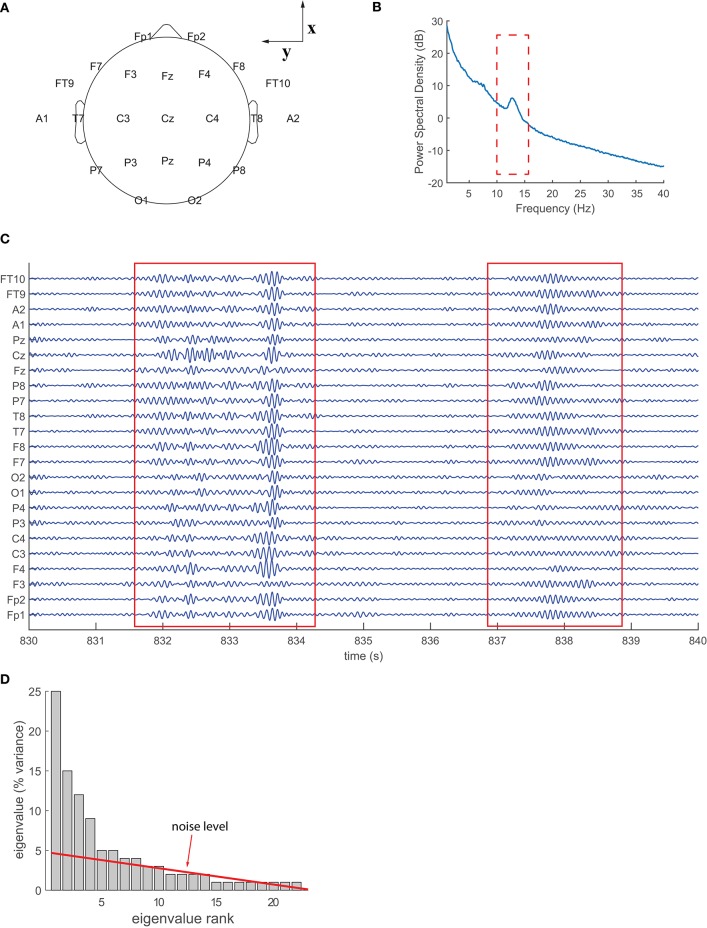
**EEG data acquisition and characteristics**. **(A)** Electrode grid and axis orientations for 10–20 system; positive **z**-axis points out of the page, through the Cz electrode. **(B)** Power spectrum for raw EEG signals averaged across channels from stage 2 non-REM sleep; a prominent peak is visible in the 10–16 Hz frequency range, corresponding to sigma waves. **(C)** Sigma-wave-filtered EEG traces; spindles are observed to be highly synchronous and present in all channels. **(D)** Eigenvalue (variance) distribution of the principal components of EEG data. The noise level is determined by extrapolating the linear trend of the tail.

EEG data were captured with the standard 10–20 system (see Methods) displayed in Figure 1A, with a total of 23 electrodes (channels). Figure 1B shows the power spectral density (PSD) of the raw EEG averaged across channels over 30 min of stage 2 non-REM sleep, for a representative normal subject. The PSD reveals a peak in the 10–16 Hz range, indicating the presence of sigma waves. These waves are prominent enough to be identified visually. Band-pass filtering in the sigma frequency band (Figure 1C) enhances their temporal profile of the canonical spindle form: recurrent, highly synchronous wavelets that wax and wane over the course of 0.5–2 s across all channels.

A principal component analysis of these filtered signals provides us with 23 components (eigenvectors of the spatial covariance matrix; see Methods) and their eigenvalues, the latter of which represent the variance carried by the associated principal component. Figure 1D displays the variance of the principal components (eigenvalues) relative to the total variance of the sigma waves (sum of all eigenvalues); the eigenvalues are ranked from largest (left) to smallest (right). Background noise contributes to the variance of each component, and the noise level can be determined empirically by linear extrapolation of the tail of the variance distribution (Mitra and Pesaran, 1999), as shown in Figure 1D (red line). The signal-to-noise ratio (SNR) for a given component is then computed as its variance divided by its noise level.

### Standing waves and spherical harmonics in normal subjects

**Figure 2:**
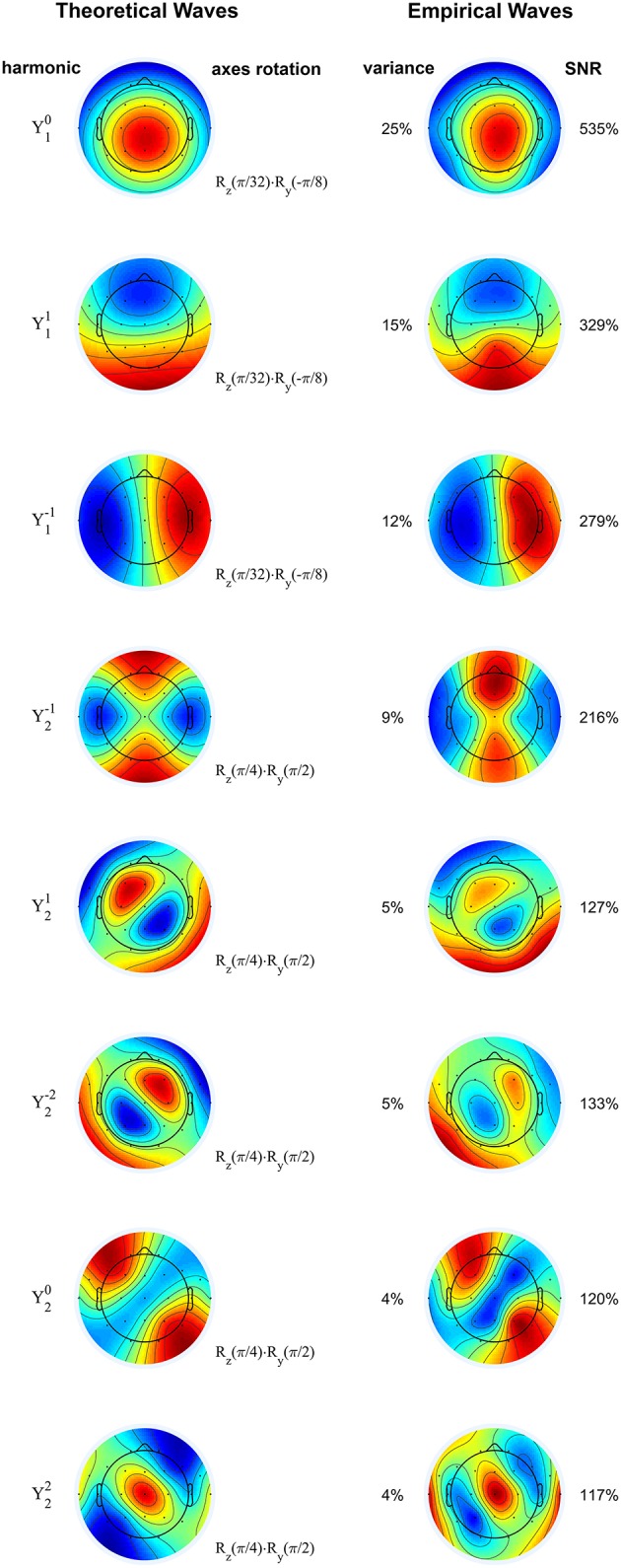
**Rotated spherical harmonics (left) and principal components of sigma waves (right) from a representative normal subject**. The theoretical and empirical waves are strikingly similar, confirming that EEG spindles during stage 2 non-REM sleep are standing waves. Spherical harmonics are manually rotated according to the axes rotation operators (denoted by **R**), based on the orientation of the empirical waves. Principal components are shown from top to bottom in order of decreasing eigenvalue (percentage of total variance). SNR: signal-to-noise ratio. Blue and red correspond to opposing signs of the waves' amplitude. Color scale covers whole range from minimum to maximum amplitude.

The principal components of the sigma waves are displayed in Figure 2 (right column) for a representative subject, in comparison with standing waves on the sphere, also known as spherical harmonics (left column), as predicted by wave theory in physics (see Methods). The first eight components capture 79% of the total variance in the data, and each of these components has an SNR greater than 100%. There is a striking similarity between the theoretical and empirical waves. The orientation of the empirical waves relative to the Cartesian axes of the spherical harmonics was determined with a three-dimensional rotation (see Methods), as reported in Figure 2. The inverse rotation maps the theoretical waves onto the empirical ones, allowing us to fully appreciate their spatial similarity. Thus, by the theory outlined in “Spherical Harmonics, Principal Components of the EEG, Standing Waves” (see Methods), the electric potential over the scalp filtered in the 10–16 Hz frequency band is comprised of standing linear waves. These results are highly consistent across all 41 neurologically normal subjects considered for analysis.

### Standing waves and spherical harmonics in patients with agenesis of the corpus callosum

We conducted similar analyses on patients with hypoplasia or complete agenesis of the corpus callosum. These patients are of particular interest because they are instrumental in validating the current view that sleep spindles are thalamo-cortical oscillations: if EEG data of patients with a defective corpus callosum display similar spherical harmonics (and consequently, standing waves) to neurologically normal patients, then the waves cannot be solely generated by cortico-cortical activity, owing to the absence or malformation of inter-hemispherical connections, and they must instead be thalamo-cortical in origin.

**Figure 3:**
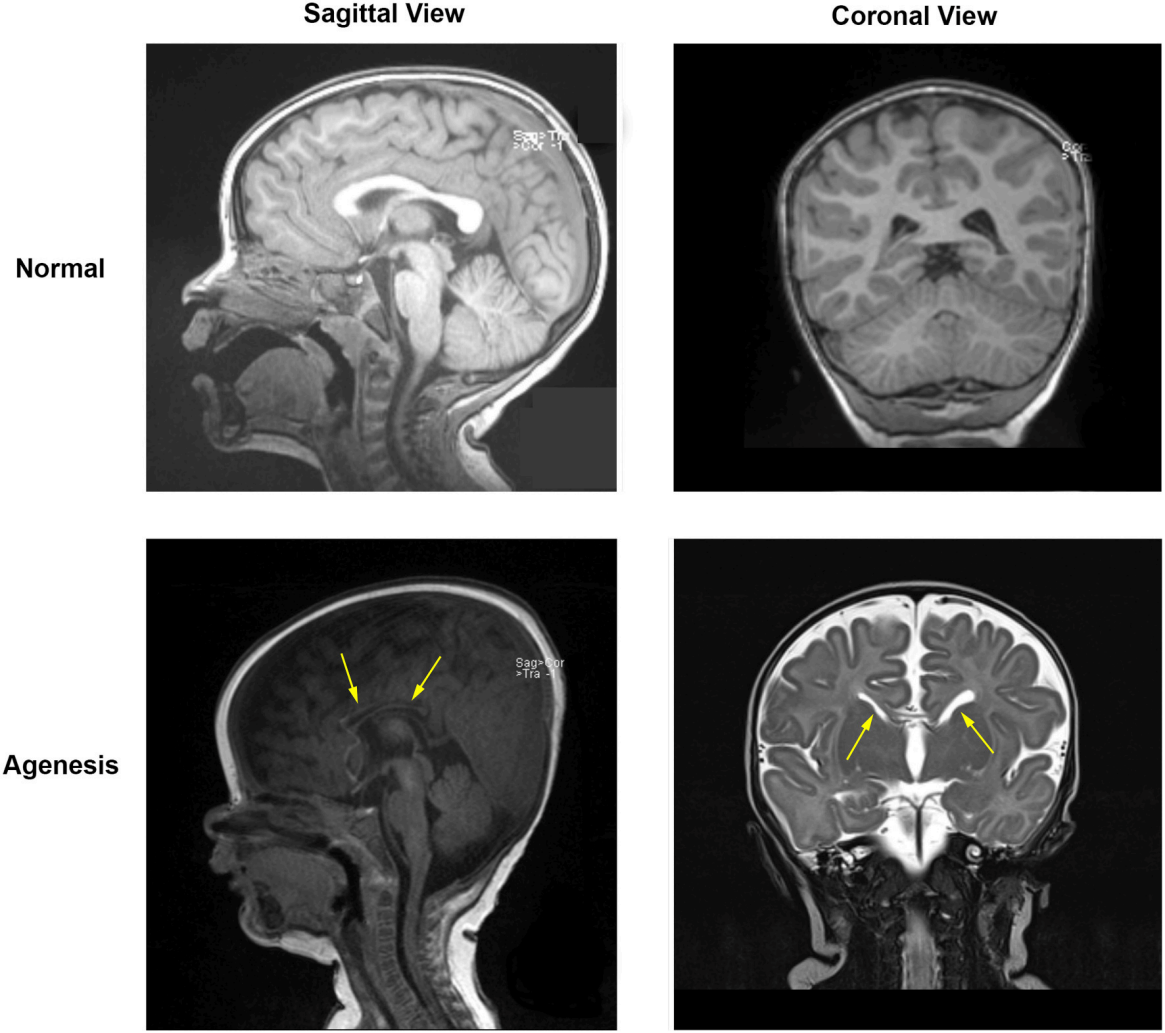
**MRI data for a normal subject (top) and for a subject with partial agenesis (bottom)**. A rift in the corpus callosum (arrows) is clearly visible in the coronal MRI image for the patient with agenesis, which is not seen in the normal subject. White matter tracts connect below and through the cortex in the normal subject, but do not do so in the patient with agenesis.

We investigated 8 patients with agenesis or hypoplasia of the corpus callosum. Two patients in this cohort each had two 30-min EEG epochs of stage 2 sleep from different dates of EEG recording; these recordings were also considered, for a total of 10 EEG recordings included in analysis. Both complete agenesis and hypoplasia of the corpus callosum are easily identified at any age by inspection of their MRI, where white matter tracts connecting the two hemispheres appear missing or underdeveloped (Figure 3).

**Figure 4:**
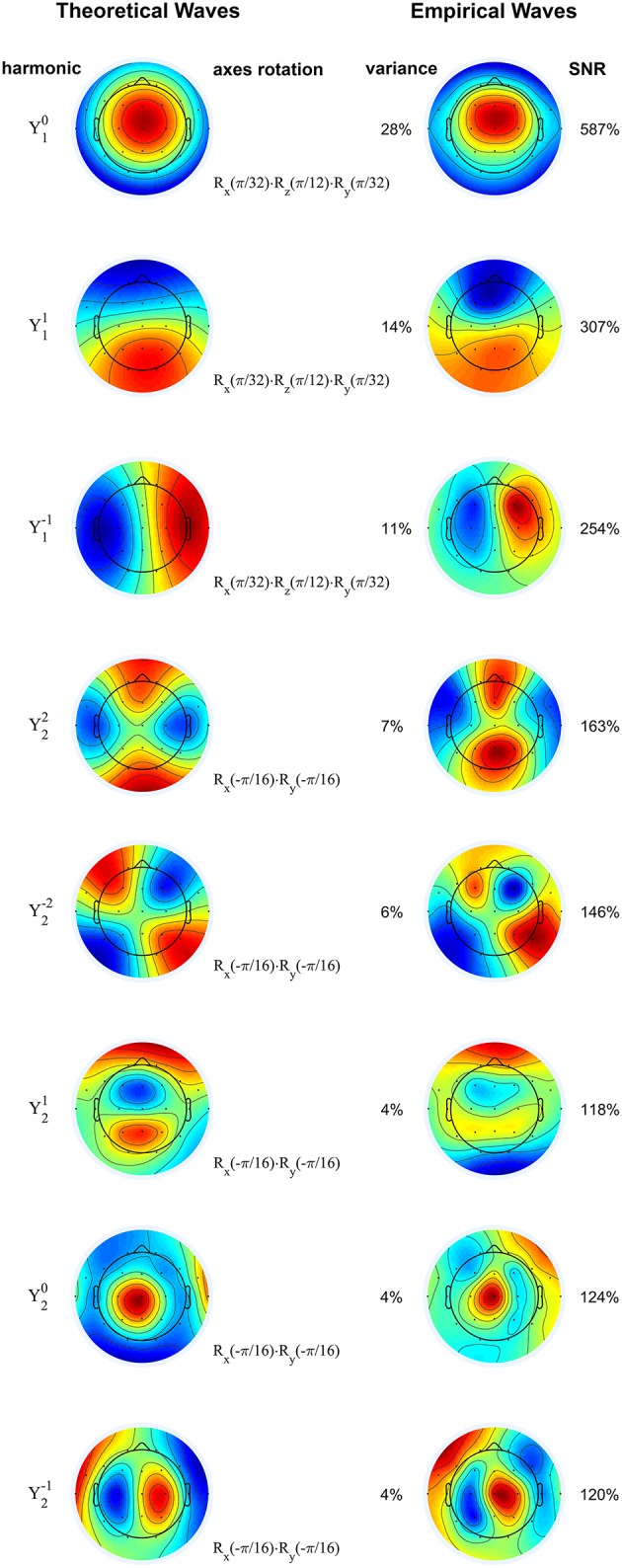
**Rotated spherical harmonics (left) and principal components of sigma waves (right) for a representative subject with agenesis of corpus callosum**. As in Figure 2, theoretical and empirical waves are strikingly similar, confirming that standing waves are also present in patients with defective inter-hemispherical connections. Spherical harmonics are manually rotated according to the axes rotation operators (denoted by **R**), based on the orientation of the empirical waves. Principal components are shown from top to bottom in order of decreasing eigenvalue (percentage of total variance). SNR: signal-to-noise ratio. Blue and red correspond to opposing signs of the waves' amplitude. Color scale covers whole range from minimum to maximum amplitude.

In accordance with the hypothesis that sigma waves are thalamo-cortical oscillations, we demonstrate in Figure 4 that the first eight principal components (each of which has an SNR greater than 100%, and which, together, capture 78% of the total variance) of the waves from a representative patient with complete agenesis of the corpus callosum are similar to the dipolar and quadrupolar spherical harmonics as predicted by theory, indicating that sigma waves exist spatiotemporally as standing waves, generally consistent with the results from normal patients. For some patients in the agenesis cohort, such as the one shown here, there appear to be some minor deviations from this trend. For example, the third component from the top depicted in Figure 4, corresponding to spherical harmonic $Y_{1}^{-1}$, is more concentrated in the “northern” hemisphere (***z*** > 0) than in the “southern” hemisphere (***z*** < 0), resembling a hemispherical harmonic. However, the spherical harmonic structure is mostly preserved in all patients with agenesis or hypoplasia of the corpus callosum, lending support to the current view that spindles are truly thalamo-cortical oscillations.

### Covariance and inverse relative distance

**Figure 5:**
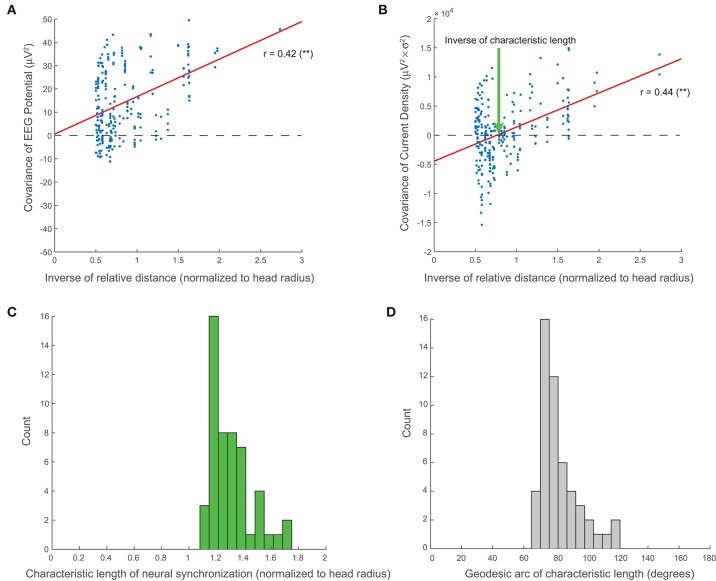
**Large-scale neural synchronization**. **(A)** As predicted by our theory (see Methods), the covariance of the EEG potential is proportional to the inverse of the relative distance. This proportionality is direct, i.e., the abscissa intercept of regression is essentially zero. **(B)** Empirically, the covariance of the current sources also shows a similar correlation with inverse distance, but with an additional horizontal offset which represents the inverse of the characteristic length of large-scale neural synchronization (normalized to the head radius). **(C)** Across all subjects, the characteristic length, *D*, clusters tightly around a mean of 1.31 (normalized to the head radius). **(D)** The angular length, or geodesic arc of the characteristic length, γ, clusters around a mean of 82.4°.

As demonstrated in Methods, the existence of standing waves implies that the spatial covariance of the EEG decreases with the inverse of the relative distance. In agreement with this prediction, Figure 5A shows that the covariance between two EEG channels is significantly correlated (*r* = 0.42, *p* = 2e-12, Pearson's correlation) with the inverse of their relative distance. Notably, the abscissa intercept of the linear regression is essentially zero, confirming the direct proportionality of $C\left(\vec{x},\vec{y}\right)$ and $1∕|\vec{x}-\vec{y}|$, as predicted by our theory. Data from all other subjects in both the normal and agenesis cohorts exhibited similar trends, with no significant differences or outliers.

Additionally, we show that the covariance of the current density on the scalp, $K\left(\vec{x},\vec{y}\right)$, appears to decay similarly with the inverse of the relative distance (*r* = 0.44, *p* = 2e-13, Pearson's correlation), but that it does so with a negative offset, β (Figure 5B). The inverse of the abscissa intercept of regression, *D* = α ∕ β, then represents the characteristic length of large-scale current source synchronization on the scalp (see “Characteristic Length of Large-Scale Neural Synchronization” in Methods). The values of *D* are clustered around a mean of 1.31 with a standard deviation of 0.15 (normalized to the head radius) for all 51 EEG recordings included in our analyses (Figure 5C). The geodesic arc γ associated with characteristic length *D* clusters around a mean of 82.4° with a standard deviation of 12.6° (Figure 5D).

## Discussion

### Summary

We have provided five major results on the interpretation of principal components of large-scale, spontaneous brain activity: (1) the spatial maps of the principal components of spontaneous EEG traces are conformal to spherical harmonics if standing waves are present; (2) the covariance of the electric potential for standing waves decays with the inverse of the relative distance; (3) empirically, a similar trend holds for the covariance of the current densities, but with an additional negative offset, leading to a finite characteristic length of large-scale neural synchronization for standing EEG waves; (4) sigma waves, or EEG spindles, during stage 2 non-REM sleep exist as standing waves, and confirm our theoretical predictions; and, (5) EEG spindles are coherent, thalamo-cortical oscillations as they also exist in patients with absence or underdevelopment of inter-hemispherical cortical networks, suggesting that they are driven and spatially coordinated by the thalamus.

### Validation of proposed neural mass models

Theoretical and computational neural-mass models, based on the principle of global fields of synaptic action, have predicted the presence of standing EEG waves for several decades (Nunez, 1981), but we believe that we are the first to show standing waves in the form of spherical harmonics of the electric potential. Inspired by mathematical discoveries in geophysics and meteorology, we have expanded the theoretical framework for interpreting principal component analyses of spatiotemporal signals. These theoretical extensions enable us to interpret the spherical harmonics observed in the principal components of EEG recordings as the presence of standing waves. The regime of stage 2, non-REM sleep provides an ideal context to corroborate our theoretical predictions. Sleep spindles are known to have high temporal synchrony across all areas of the cortex (Huguenard and McCormick, 2007), as exemplified in Figure 1C, and therefore are more likely to display large-scale spatial covariations over time, which most readily manifest as spherical harmonics over a domain conformal to a sphere.

Incidentally, we have also observed spherical harmonics in the principal components of delta waves (~1 Hz). However, because our EEG clips are only from stage 2 sleep, delta waves contribute little to the EEG signals and are scarce in many subjects (data not shown). So, we focused on the sigma waves, which clearly dominate the EEG signals across all subjects.

### Lack of degeneracy and symmetry breaking in empirical spherical harmonics

Spherical harmonics appear in the solution to equations involving the Laplacian in systems with spherical symmetry, as is the case of Schrödinger's equation in quantum mechanics for electrons in hydrogenic atoms, in which the potential energy only depends on the distance to the nucleus. This application of quantum theory places emphasis on the concept of degeneracy of harmonics, whereby several harmonics may share the same energy (Atkins and Friedman, 2011). The angular part of the electronic wavefunction $Y_{l}^{m}$ is degenerate for given *l* and all integers *m* with −*l* ≤ *m* ≤ *l*, but the energy levels of these harmonics may be split by breaking the spherical symmetry of the system, for example, by applying an external electric field to the atom, known as the Stark effect (Atkins and Friedman, 2011). The concept of degeneracy similarly applies to the principal components of EEG waves: the relative contribution of each component in a set representing degenerate harmonics is expected to contribute the same amount to the total variance of the system, i.e., have the same eigenvalues. However, from Figure 1D, it is clear that the components, including the simplest cases of dipoles (*n* = 1, 2, 3) and quadrupoles (*n* = 4, 5, …, 8), do not have the same eigenvalues, and thus we may conclude that the spherical symmetry of the standing wave model is broken.

The symmetry breaking we observe can be attributed to at least two aspects of scalp EEG recordings. The first aspect is the effect of uneven sampling across the head: EEG electrodes sample the northern hemisphere more thoroughly than the southern hemisphere, and thus place greater emphasis on signals with similar spatial bias. Considering the power and synchrony of sigma waves (visible in Figures 1B,C, respectively) during stage 2 non-REM sleep, spherical extrapolation of the already synchronous northern hemisphere is most likely to accentuate the ***z***-axis dipolar component ($Y_{1}^{0}$) in spatiotemporal principal component decomposition, thereby increasing its eigenvalue and breaking degeneracy with other dipolar components.

A second aspect contributing to symmetry breaking is the effect of the non-spherical shapes of the human brain and head. Indeed, geometrical differences between the head and an actual sphere represent perturbations to the Laplacian in a mathematical sense, and are therefore expected to alter the distribution of its eigenvalues, as well as the degeneracy of its principal components. The effect of the head's shape on the eigenvalues of the Laplacian is reminiscent of an exciting problem in mathematical physics: can one hear the shape of a drum? (Kac, 1966).

### Orientation of spherical waves and its possible interpretation

In addition to degeneracy arising from symmetry breaking, principal components also display rotational differences, which must be empirically determined (see Methods). These deviations indicate that although brain activity during stage 2 non-REM sleep may be effectively characterized using spherical harmonics, their orientations may conceal information that differs from individual to individual, and between different phenotypes. Analyses of the rotations of these harmonics in future studies may lead to finer understanding of the origins of sleep spindles and other relevant EEG waves, as they could be related to specific thalamo-cortical projections or excitatory-inhibitory networks in the cortex. It is also possible that these rotations co-localize with functional structures in the brain, such as white matter tracts.

### Analogy with quantum mechanics: “thermodynamic” equilibrium and consciousness

Non-REM sleep is considered to be the period when an individual loses consciousness (Hobson and Pace-Schott, 2002), stage 2 non-REM is when gamma waves (which correlate with conscious brain activity) begin to disappear (Balduzzi et al., 2008), and sleep spindles are known to be integral to basic cognitive functions such as memory consolidation (Marshall and Born, 2007). An analogy of stage 2 spherical waves with statistical and quantum mechanics provides an interesting framework to approach the challenging problem of consciousness. First, we recall that the relative variance of a principal component of spontaneous neural activity, i.e., its normalized eigenvalue, can be interpreted as a probability (Galán, 2008). For instance, if a given harmonic is produced by the synchronous activity of *n* neurons out of a very large total of *N* neurons, the relative weight of its associated eigenvalue can be thought of as the ratio *n*∕*N*. Thus, the eigenvalue distribution of the spherical waves after proper normalization is akin to a Boltzmann distribution, which, by definition, represents a state of “thermodynamic” equilibrium. It follows that transitions to the awake, conscious state, or to the dreaming state observed in REM sleep, represent significant deviations from this equilibrium.

The idea that consciousness is a manifestation of electromagnetic fields generated by neural activity and, reciprocally, that consciousness affects neural activity, has been previously entertained by other authors, with different flavors. Libet, in his theory of the conscious mental field, noted that consciousness also affects neural activity, and proposed that electromagnetic fields provide the link between mind and neural activity, and that the former is an emergent property of the latter (Libet, 2004). McFadden, in his theory of conscious electromagnetic information (McFadden, 2002, 2013), suggests that consciousness is the component of the electromagnetic field that feeds back to modulate neural activity, a view that is shared by Fingelkurts' brain-mind operational architectonics theory (Fingelkurts and Fingelkurts, 2004).

In their recent relativistic brain theory, Cicurel and Nicolelis argue that the brain works as an analog computer rather than a digital one (Turing machine), and that analog computations involve electromagnetic fields generated by white matter loops, or “coils” (Cicurel and Nicolelis, 2015). The fluctuations of these fields are continuous in both time and space, and they change in response to external stimuli in a state-dependent manner, i.e., relative to the current state of the brain (hence the adjective “relativistic”). These electromagnetic fields in turn constrain and “glue” the firing of neurons (observed as spikes), defining a mental space in which higher-order functions, including consciousness, emerge. Consistent with this view, our theory and experimental data show that the electric potential of the EEG during unconscious, state 2 sleep oscillates in thalamo-cortical loops (“coils”) spontaneously, adopting highly reproducible configurations, which represent a true resting state of the brain. As explained above, this is analogous to the thermodynamic equilibrium in statistical and quantum mechanics, and suggests that transitions to a conscious state represent deviations from this equilibrium.

## Methods

### Human subjects and patient selection

Retrospective chart review and data collection from the EEG database of the Pediatric Epilepsy Unit at University Hospitals were both approved by the Institutional Review Board at Case Western Reserve University and University Hospitals of Cleveland, as part of a broader project to investigate EEG activity patterns in children and adolescents with neurological conditions. This study concerns two groups of subjects: 41 neurologically normal controls, and 8 patients with agenesis or hypoplasia of the corpus callosum. Two subjects of the latter group had EEG recordings from two different dates of admission, making up a total of 10 EEG recordings in the agenesis cohort. Of the normal subjects, 14 (34%) were male and 27 (66%) were female, with ages ranging from 9 months to 18 years; of the subjects with agenesis or hypoplasia of the corpus callosum, 3 (37%) were male and 5 (63%) were female, with ages ranging from 3 months to 14 years.

### EEG registration

EEG recordings were collected from subjects during overnight sleep observations (8–12 h) at the Pediatric Epilepsy Unit and used the standard 10–20 electrode system at a sampling frequency of 200 Hz, including the 23 electrodes shown in Figure 1A: Fp1, Fp2, F7, F3, Fz, F4, F8, FT9, FT10, A1, T7, C3, Cz, C4, T8, A2, P7, P3, Pz, P4, P8, O1, and O2. Electrodes were referenced to a ground electrode placed on the center of the forehead (Fpz) and all analyses were performed on EEG waveforms referenced to this common electrode. After data acquisition, 30 min of uninterrupted stage 2 non-REM sleep were identified by a hospital technician using standard criteria: presence of sleep spindles and K-complexes, and fewer than 4 s of delta waves (0–4 Hz) in a “page” of 15 s of EEG, in the bipolar double-banana montage. In the case that multiple 30-min epochs of stage 2 non-REM sleep were available from the same observation, the chronologically first one was selected. Electrode impedances were all maintained at or below 5 kΩ by means of a surface electrode gel optimized for long-term monitoring applications, throughout the 30 min of stage 2 non-REM sleep. These 30-min epochs were clipped from each EEG recording for the subsequent analyses. All the analyzed EEG clips consisted of high-quality recordings only and contained no artifacts.

### Signal processing

Analyses of EEG signals were performed in MATLAB Academic Version 2015a. To investigate sigma waves, signals from each electrode were mean-subtracted and filtered offline both forward and backward in time (using MATLAB's *filtfilt* function) with a sixth-order Butterworth, band-pass filter between 10 and 16 Hz. Principal components were computed as the eigenvectors of the covariance matrix of the filtered signals and ranked from highest to lowest according to their eigenvalues. Spatial maps of the principal components were displayed using the *topoplot* function of the EEGLAB software package, version 13.4.4b (Delorme and Makeig, 2004).

### Definition of spherical harmonics

The Laplacian, Δ, is a differential operator defined as the divergence of the gradient of a scalar function, which in Cartesian coordinates takes the form

$$\begin{array}{l} \Delta f\left(\vec{x}\right)\equiv \nabla \cdot \nabla f\left(\vec{x}\right)=\left(\frac{\partial^{2}}{\partial x_{1}^{2}}+\frac{\partial^{2}}{\partial x_{2}^{2}}+\frac{\partial^{2}}{\partial x_{3}^{2}}\right)f\left(\vec{x}\right). \end{array}$$

By definition, spherical harmonics are the eigenfunctions of the Laplacian on a spherical surface, that is, any function function $u\left(\vec{x}\right)$ which satisfies

(1) $$\begin{array}{l} \Delta u\left(\vec{x}\right)=-\mu u\left(\vec{x}\right), \end{array}$$

where −μ is its associated eigenvalue, and the negative sign is taken by convention to emphasize that it is a non-positive number, and hence μ is non-negative. Using spherical coordinates, the eigenvalues are given by μ = *l*(*l* + 1), where *l* is a non-negative integer: *l* = 0, 1, 2, … For a given value of *l*, there are 2*l* + 1 eigenfunctions, one for each integer *m* with −*l* ≤ *m* ≤ *l*. Each possible pair of *m* and *l* then determines an *n*-th eigenfunction, or spherical harmonic, given by

$$u_{n}(\vec{x})=Y_{l}^{m}(\theta ,\varphi )=\{\begin{array}{l} \sqrt{\frac{\left(2l\;+\;1\right)}{2\pi }\frac{\left(l\;-\;\left|m\right|\right)!}{\left(l\;+\;\left|m\right|\right)!}}P_{l}^{\left|m\right|}\left(\cos(\theta )\right)\sin\left(\left|m\right|\varphi \right),\\ \text{ if }m<0\\ \sqrt{\frac{\left(2l\;+\;1\right)}{4\pi }}P_{l}^{m}\left(\cos(\theta )\right),\\ \text{ if }m=0\\ \sqrt{\frac{\left(2l\;+\;1\right)}{2\pi }\frac{\left(l\;-\;m\right)!}{\left(l\;+\;m\right)!}}P_{l}^{m}\left(\cos(\theta )\right)\cos\left(m\varphi \right),\\ \text{ if }m>0 \end{array}$$

where $P_{l}^{m}\left(x\right)$ is the associated Legendre polynomial of degree *l* and order *m*; θ is the polar, or zenith, angle; and φ is the longitude, or azimuth.

### Spatial rotation of spherical harmonics

The theoretical spherical harmonics above are defined relative to the EEG's Cartesian axes, **x,y,z**, as displayed in Figure 1A. By convention, x points through the nasion (positive values toward the anterior of the head), *y* through the left pre-auricular point (positive values toward the left ear), and *z* directly upwards (positive values toward the superior direction) through the Cz electrode position. However, the orientation of these axes may not coincide with the orientation of the axes **x**′,**y**′,**z**′, with respect to which the empirical spherical harmonics are aligned. For the purpose of comparing theoretical and empirical waves, as shown in Figures 2, 4, one may need to transform the theoretical axes to align them with the empirical ones. This is accomplished through at most one rotation around each of the Cartesian axes. It should be noted that the order of the axes rotations matters, as these operations do not commute. Without loss of generality, let us assume that the required transformation consists of a sequence of rotations around the **x,y,z** axes, of angles ω_**x**_, ω_**y**_, ω_**z**_, respectively. The transformation of the axes is then given by

$$\begin{array}{l} \left[\begin{array}{c} {\text{x}}^{\prime }\\ {\text{y}}^{\prime }\\ {\text{z}}^{\prime } \end{array}\right]={\text{R}}_{\text{z}}\left(\omega_{\text{z}}\right)\cdot {\text{R}}_{\text{y}}\left(\omega_{\text{y}}\right)\cdot {\text{R}}_{\text{x}}\left(\omega_{\text{x}}\right)\left[\begin{array}{c} \text{x}\\ \text{y}\\ \text{z} \end{array}\right], \end{array}$$

where the rotation matrices **R**_**x**_, **R**_**y**_, **R**_**z**_ are defined as

$$\begin{array}{l} \begin{array}{c} {\text{R}}_{\text{x}}\left(\omega_{\text{x}}\right)=\left[\begin{array}{ccc} 1 & 0 & 0\\ 0 & \cos\omega_{\text{x}} & -\sin\omega_{\text{x}}\\ 0 & -\sin\omega_{\text{x}} & \cos\omega_{\text{x}} \end{array}\right],\\ {\text{R}}_{\text{y}}\left(\omega_{\text{y}}\right)=\left[\begin{array}{ccc} \cos\omega_{\text{y}} & 0 & \sin\omega_{\text{y}}\\ 0 & 1 & 0\\ -\sin\omega_{\text{y}} & 0 & \cos\omega_{\text{y}} \end{array}\right],\\ {\text{R}}_{\text{z}}\left(\omega_{\text{z}}\right)=\left[\begin{array}{ccc} \cos\omega_{\text{z}} & -\sin\omega_{\text{z}} & 0\\ \sin\omega_{\text{z}} & \cos\omega_{\text{z}} & 0\\ 0 & 0 & 1 \end{array}\right]. \end{array} \end{array}$$

In practice, we do not rotate the theoretical axes, but transform the coordinates of the harmonics themselves. To do this, we perform the inverse transformation ${\text{R}}_{\text{x}}^{-1}\left(\omega_{\text{x}}\right)\cdot {\text{R}}_{\text{y}}^{-1}\left(\omega_{\text{y}}\right)\cdot {\text{R}}_{\text{z}}^{-1}\left(\omega_{\text{z}}\right)$ on the coordinates of their maps.

### Spherical harmonics, principal components of the EEG, standing waves

We demonstrate here that the principal components of EEG spindles represent standing waves and are equivalent to spherical harmonics. Let $V\left(\vec{x},t\right)$ be the mean-subtracted electric potential of the EEG at position $\vec{x}$ on the scalp at time *t*. The stationary covariance of two potentials recorded at locations $\vec{x}=\left(x_{1},x_{2},x_{3}\right)$ and $\vec{y}=\left(y_{1},y_{2},y_{3}\right)$ on the head's surface is defined as their product averaged in time:

(2) $$\begin{array}{l} C\left(\vec{x},\vec{y}\right)=\underset{T\to \infty }{\lim}\frac{1}{T}\overset{T}{\underset{0}{\int }}V\left(\vec{x},t\right)V\left(\vec{y},t\right)dt\equiv \langle V\left(\vec{x},t\right)V\left(\vec{y},t\right)\rangle . \end{array}$$

On the head's surface, which conforms to a spherical surface *S*, the covariance operator acting on an arbitrary function of space $f\left(\vec{y}\right)$ is defined as

(3) $$\begin{array}{l} \underset{S}{\int }C\left(\vec{x},\vec{y}\right)f\left(\vec{y}\right)dS\left(\vec{y}\right), \end{array}$$

where $dS\left(\vec{y}\right)$ denotes integration over *S* with respect to $\vec{y}$. Note that the covariance operator is an integral operator.

By definition, the principal components are the eigenfunctions of the covariance operator, that is, any function $v\left(\vec{x}\right)$ which satisfies

$$\begin{array}{l} \underset{S}{\int }C\left(\vec{x},\vec{y}\right)v\left(\vec{y}\right)dS\left(\vec{y}\right)=\lambda v\left(\vec{x}\right), \end{array}$$

where λ is its associated eigenvalue representing the signal's variance carried by that principal component. If none of the eigenvalues are repeated or equal to zero (as is typically the case for the empirical covariance operator due to the presence of noise in the recorded signals), there are as many principal components *N* as electrodes and they form an orthogonal basis, so that any spatial function on *S* can be expanded on that basis. In particular, for the electric potential at time *t*, one has

(4) $$\begin{array}{l} V\left(\vec{x},t\right)=\underset{n}{\sum }a_{n}\left(t\right)v_{n}\left(\vec{x}\right), \end{array}$$

with $a_{n}\left(t\right)=\underset{S}{\int }v_{n}\left(\vec{x}\right)V\left(\vec{x},t\right)dS\left(\vec{x}\right)$, and *n* = 1, 2, …, *N*. The time series *a*_*n*_(*t*) are uncorrelated with each other:

$$\begin{array}{l} \langle a_{n}\left(t\right)a_{m}\left(t\right)\rangle =\underset{S}{\int }\underset{S}{\int }v_{n}\left(\vec{x}\right)\langle V\left(\vec{x},t\right)V\left(\vec{y},t\right)\rangle v_{m}\left(\vec{y}\right)dS\left(\vec{x}\right)dS\left(\vec{y}\right)\\ =\underset{S}{\int }\underset{S}{\int }v_{n}\left(\vec{x}\right)C\left(\vec{x},\vec{y}\right)v_{m}\left(\vec{y}\right)dS\left(\vec{x}\right)dS\left(\vec{y}\right)\\ =\underset{S}{\int }v_{n}\left(\vec{x}\right)\underset{S}{\int }C\left(\vec{x},\vec{y}\right)v_{m}\left(\vec{y}\right)dS\left(\vec{x}\right)dS\left(\vec{y}\right)\\ =\lambda_{m}\underset{S}{\int }v_{n}\left(\vec{x}\right)v_{m}\left(\vec{x}\right)dS\left(\vec{x}\right)\\ =\lambda_{m}\delta_{nm}, \end{array}$$

where δ_*nm*_ is the Kronecker delta function: δ_*nm*_ = 1, if *n* = *m*; and δ_*nm*_ = 0, if *n* ≠ *m*.

It is well-known in linear algebra that two linear operators have the same eigenfunctions if, and only if, they commute (Horn and Johnson, 1985), although the associated eigenvalues may be different. Thus, the principal components will be identical with the spherical harmonics if, and only if, the covariance operator commutes with the Laplacian. Mathematically, this is represented as

(5) $$\begin{array}{l} \Delta_{\vec{x}}\underset{S}{\int }C\left(\vec{x},\vec{y}\right)u\left(\vec{y}\right)dS\left(\vec{y}\right)=\underset{S}{\int }C\left(\vec{x},\vec{y}\right)\Delta_{\vec{y}}u\left(\vec{y}\right)dS\left(\vec{y}\right), \end{array}$$

where the subscript of the Laplacian denotes the coordinates with respect to which we differentiate. Then, by replacing (2) in (5), we have

$$\begin{array}{l} \underset{S}{\int }\langle \Delta_{\vec{x}}V\left(\vec{x},t\right)V\left(\vec{y},t\right)\rangle u\left(\vec{y}\right)dS\left(\vec{y}\right)=\underset{S}{\int }\langle V\left(\vec{x},t\right)V\left(\vec{y},t\right)\rangle \Delta_{\vec{y}}u\left(\vec{y}\right)dS\left(\vec{y}\right). \end{array}$$

Including the time-independent factors in the temporal average, we obtain

$$\begin{array}{l} \underset{S}{\int }\langle \Delta_{\vec{x}}V\left(\vec{x},t\right)V\left(\vec{y},t\right)u\left(\vec{y}\right)\rangle dS\left(\vec{y}\right)=\underset{S}{\int }\langle V\left(\vec{x},t\right)V\left(\vec{y},t\right)\Delta_{\vec{y}}u\left(\vec{y}\right)\rangle dS\left(\vec{y}\right). \end{array}$$

This equation will hold in general if the integrands of both sides are equal, which leads us to

$$\begin{array}{l} \Delta_{\vec{x}}V\left(\vec{x},t\right)V\left(\vec{y},t\right)u\left(\vec{y}\right)=V\left(\vec{x},t\right)V\left(\vec{y},t\right)\Delta_{\vec{y}}u\left(\vec{y}\right). \end{array}$$

Simplifying the common factor, we obtain

$$\begin{array}{l} \Delta V\left(\vec{x},t\right)u\left(\vec{y}\right)=V\left(\vec{x},t\right)\Delta u\left(\vec{y}\right). \end{array}$$

On each side, we now regroup factors with the same variables:

$$\begin{array}{l} \frac{\Delta V\left(\vec{x},t\right)}{V\left(\vec{x},t\right)}=\frac{\Delta u\left(\vec{y}\right)}{u\left(\vec{y}\right)}. \end{array}$$

This equation will be satisfied for any $\vec{x}$ and $\vec{y}$ if each side is constant, say, equal to −μ:

$$\begin{array}{l} \frac{\Delta V\left(\vec{x},t\right)}{V\left(\vec{x},t\right)}=\frac{\Delta u\left(\vec{y}\right)}{u\left(\vec{y}\right)}=-\mu , \end{array}$$

which leads to two equations. One is the eigenvalue problem for the Laplacian

(6) $$\begin{array}{l} \Delta u\left(\vec{y}\right)+\mu u\left(\vec{y}\right)=0, \end{array}$$

which is the same as Equation (1). The other is $\Delta V\left(\vec{x},t\right)+\mu V\left(\vec{x},t\right)=0$, which, after factorizing the electric potential into a time-dependent function and a space-dependent function, $V\left(\vec{x},t\right)=U\left(\vec{x}\right)\cdot T\left(t\right)$, leads to the so-called Helmholtz's equation for a given μ:

(7) $$\begin{array}{l} \Delta U\left(\vec{x}\right)+\mu U\left(\vec{x}\right)=0. \end{array}$$

Equations (6) and (7) are formally identical, so they have the same solution. The solution to Helmholtz's equation represents a stationary, or standing, linear wave, whose amplitude profile $U\left(\vec{x}\right)$ is modulated in time by *T*(*t*) without propagating. In conclusion, if the recorded electric potential $V\left(\vec{x},t\right)$ represents a standing wave, the covariance operator and the Laplacian will commute, and therefore they will have the same eigenfunctions. As a result, for an electric potential over a spherical domain (or a domain conformal to a sphere, such as the human head), the principal components will be spherical harmonics (or a conformal version of them).

### Standing waves and the covariance of the EEG

We know from linear algebra that a linear operator and its inverse operator have the same eigenfunctions. The inverse operator of the Laplacian is the Green's operator:

$$\begin{array}{l} \underset{S}{\int }G\left(\vec{x},\vec{y}\right)f\left(\vec{y}\right)dS\left(\vec{y}\right), \end{array}$$

where $G\left(\vec{x},\vec{y}\right)$ is a Green's function, satisfying

$$\begin{array}{l} \Delta_{\vec{x}}G\left(\vec{x},\vec{y}\right)=\delta \left(\vec{x}-\vec{y}\right), \end{array}$$

where δ is the Dirac delta function. For a spherical surface, the Green's function is proportional to the inverse of the relative distance:

$$\begin{array}{l} G\left(\vec{x},\vec{y}\right)=-\frac{1}{4\pi |\vec{x}-\vec{y}|}. \end{array}$$

Since the covariance operator for sigma waves commutes with the Laplacian, it should also commute with its inverse, the Green's operator. Thus, the following equality should hold:

$$\begin{array}{l} \underset{S}{\int }\frac{1}{\left|\vec{x}-\vec{u}\right|}\underset{S}{\int }C\left(\vec{u},\vec{y}\right)u\left(\vec{y}\right)dS\left(\vec{y}\right)dS\left(\vec{u}\right)\\ =\underset{S}{\int }C\left(\vec{x},\vec{u}\right)\underset{S}{\int }\frac{u\left(\vec{y}\right)}{\left|\vec{u}-\vec{y}\right|}dS\left(\vec{y}\right)dS\left(\vec{u}\right), \end{array}$$

where the factor −1 ∕ (4π) has canceled out on each side. Regrouping the integrands, one has

$$\begin{array}{l} \underset{S}{\int }\underset{S}{\int }\frac{C\left(\vec{u},\vec{y}\right)}{\left|\vec{x}-\vec{u}\right|}u\left(\vec{y}\right)dS\left(\vec{y}\right)dS\left(\vec{u}\right)=\underset{S}{\int }\underset{S}{\int }\frac{C\left(\vec{x},\vec{u}\right)}{\left|\vec{u}-\vec{y}\right|}u\left(\vec{y}\right)dS\left(\vec{y}\right)dS\left(\vec{u}\right). \end{array}$$

This equation will hold in general if the integrands of both sides are equal, which leads us to

$$\begin{array}{l} \frac{C\left(\vec{u},\vec{y}\right)}{\left|\vec{x}-\vec{u}\right|}=\frac{C\left(\vec{x},\vec{u}\right)}{\left|\vec{u}-\vec{y}\right|}. \end{array}$$

By regrouping, on each side, factors with the same variables, we obtain

$$\begin{array}{l} C\left(\vec{u},\vec{y}\right)|\vec{u}-\vec{y}|=C\left(\vec{x},\vec{u}\right)|\vec{x}-\vec{u}|. \end{array}$$

This equation will be satisfied for any $\vec{x}$, $\vec{y}$, and $\vec{u}$, if each side is constant, which implies that

$$\begin{array}{l} C\left(\vec{x},\vec{y}\right)\sim \frac{1}{\left|\vec{x}-\vec{y}\right|}. \end{array}$$

We thus, conclude that for standing waves, the covariance of EEG signals at two separate locations is proportional to the inverse of their relative distance.

### Spherical harmonics, volume conduction, and current densities

EEG signals can be physically described and modeled in terms of volume conduction (Nunez and Srinivasan, 2006a). Current densities due to neural activity propagate through the brain and the skull, which behave as resistive media, and in which capacitive and inductive effects are ignored, as they are negligible for relatively slow fluctuations of the electric fields. Thus, the volume conduction equation takes the form

$$\begin{array}{l} \nabla \cdot \left(\sigma \left(\vec{x}\right)\nabla V\left(\vec{x},t\right)\right)=-I\left(\vec{x},t\right), \end{array}$$

where $\sigma \left(\vec{x}\right)$ is the conductivity tensor, $V\left(\vec{x},t\right)$ is again the electric potential, and $I\left(\vec{x},t\right)$ is the current density (a source if positive, and a sink if negative). In standard spherical models of the human head, the conductivity tensor is considered isotropic and homogeneous within the brain, but changes in conductivity appear at the boundaries between brain, dura, skull, and skin. The volume conduction problem then reduces to 4 equations of the form

(8) $$\begin{array}{l} \sigma \Delta V\left(\vec{x},t\right)=-I\left(\vec{x},t\right), \end{array}$$

each with a different value for the conductivity. The global solution must then be properly “stitched” at the boundaries between brain, dura, skull, and skin. Here, we focus on (8) for a single domain, ignoring these boundary effects. Then, by replacing (4) in (8) and using (1), we obtain an estimation of the current density on the head's surface:

(9) $$\begin{array}{l} I\left(\vec{x},t\right)=-\sigma \underset{n}{\sum }a_{n}\left(t\right)\mu_{n}v_{n}\left(\vec{x}\right). \end{array}$$

This series expansion converges more slowly than the series for the electric potential (4), because although the variance of the time series *a*_*n*_(*t*) decreases roughly exponentially with *n*, the non-degenerate eigenvalues of the Laplacian μ_*n*_ grow monotonically with *n*. For instance, μ_*n*_ = 2 for dipolar harmonics, and μ_*n*_ = 6 for quadrupolar harmonics. We also note that because of the limited spatial resolution of the standard EEG 10–20 electrode grid, harmonics of higher order cannot be adequately resolved, so the series expansion (9) must be truncated up to the quadrupolar order.

From (4), the covariance of the EEG potential can be expressed as a function of the principal components of the sigma waves:

$$\begin{array}{l} \langle V\left(\vec{x},t\right)V\left(\vec{y},t\right)\rangle =\underset{n}{\sum }\underset{m}{\sum }\langle a_{n}\left(t\right)a_{m}\left(t\right)\rangle v_{n}\left(\vec{x}\right)v_{m}\left(\vec{y}\right)\\ =\underset{n}{\sum }\lambda_{n}^{2}v\left(\vec{x}\right)v\left(\vec{y}\right). \end{array}$$

Similarly, from (9), the covariance of the current densities on the head's surface can be expressed as

$$\begin{array}{l} K\left(\vec{x},\vec{y}\right)=\langle I\left(\vec{x},t\right)I\left(\vec{y},t\right)\rangle =\sigma^{2}\underset{n}{\sum }\underset{m}{\sum }\langle a_{n}\left(t\right)a_{m}\left(t\right)\rangle \mu_{n}^{2}v_{n}\left(\vec{x}\right)v_{m}\left(\vec{y}\right)\\ =\sigma^{2}\underset{n}{\sum }\lambda_{n}^{2}\mu_{n}^{2}v\left(\vec{x}\right)v\left(\vec{y}\right). \end{array}$$

In our calculations we normalize the current densities to the conductivity, and their spatial covariance to the conductivity squared, that is, we consider σ = 1.

### Characteristic length of large-scale neural synchronization

We have shown above that the covariance of the EEG potential decays with the inverse of the relative distance. Empirically, the covariance of the current densities follows a similar trend but with an additional, negative offset:

$$\begin{array}{l} K\left(\vec{x},\vec{y}\right)\sim \frac{\alpha }{\left|\vec{x}-\vec{y}\right|}-\beta , \end{array}$$

with both α, β > 0. This indicates that there is a finite distance, *D* = α ∕ β, for which the covariance of the current densities is zero. This distance represents the *characteristic length* of large-scale neural synchronization: for smaller distances, the current densities will be synchronized (positively correlated), so that at both locations $\vec{x}$ and $\vec{y}$ there are current sources (or sinks) at the same time; for larger distances, the current densities are negatively correlated, so that when there is a source at $\vec{x}$, there will be a sink at $\vec{y}$, and vice versa. The angular distance, or equivalently, the geodesic arc expressed in radians, for the characteristic length is then given by

$$\begin{array}{l} \gamma =\text{acos}\left(1-\frac{D^{2}}{2\rho^{2}}\right), \end{array}$$

where ρ is the radius of the spherical head model, and 0 ≤ γ ≤ π. For comparison purposes, we normalize the radius for each subject to ρ = 1 in Figures 5A–C.

## Author contributions

RFG conceived and designed the research study. AGN compiled and analyzed the clinical reports. AGN, SSS, and RFG collected the EEG data. SSS and RFG analyzed the data, generated the figures, and wrote the manuscript. All authors approved the final version of the manuscript.

## Funding

This work has been supported by a Biomedical Researcher Award of The Hartwell Foundation (RFG).

### Conflict of interest statement

The authors declare that the research was conducted in the absence of any commercial or financial relationships that could be construed as a potential conflict of interest.
